# Neuroepidemiology of Post‐Stroke Dementia in a Sub‐Saharan African Population

**DOI:** 10.1002/alz70860_103792

**Published:** 2025-12-23

**Authors:** Mundih N Njohjam, Niakam F Tiffany, Mark O Ngoule, Annick‐Sandra J Teugang, Alex Cyrille C Mondomobe

**Affiliations:** ^1^ National University Teaching Hospital, Dakar, United Kingdom; ^2^ Department of Neurology, Cheikh Anta Diop University, Dakar, Dakar, Senegal; ^3^ National University Teaching Hospital, Dakar, Dakar, Senegal

## Abstract

**Background:**

The neuroepidemiological and clinical characteristics of post‐stroke dementia (PSD) in Sub‐Saharan African populations have not been sufficiently described. The objective of this study was to determine the prevalence, risk factors, and clinical presentation of PSD in a sub‐Saharan African population of stroke survivors.

**Method:**

Cognitive impairment was assessed using the Montreal Cognitive Assessment (MoCA), Dubois' five‐word test, and the frontal assessment battery. Post‐stroke dementia was diagnosed using the National Institute of Neurological Disorders and Stroke‐Association Internationale pour la Recherche et Enseignement en Neurosciences (NINDS‐AIREN) criteria. The clinical dementia rating scale (CDR) was used to assess and stage the severity of PSD.

**Result:**

A total of 412 stroke survivors were screened, of whom 78 met the criteria for PSD with an age range between 38 and 84, giving a prevalence of 18.9%. Factors associated with PSD included silent brain infarctions, markers of small vessel disease, atrial fibrillation, age, and level of education. Multi‐infarct PSD was the most common type. The most affected cognitive domain was executive functions, and most (74.9%) PSD patients had severe PSD based on CDR. None of the patients was receiving dementia‐specific care at the time of diagnosis.

**Conclusion:**

PSD is a growing public health problem in Sub‐Saharan African populations.

